# Rebamipide Attenuates Lupus Nephritis by Enhancing Antioxidative Defense in Podocytes: Evidence from a Lupus-Prone Mouse Model

**DOI:** 10.3390/ijms26125809

**Published:** 2025-06-17

**Authors:** Young-Suk Song, Youngjae Park, Da-Som Kim, Se Gwang Jang, Seung-Ki Kwok

**Affiliations:** 1 The Rheumatism Research Center, Catholic Research Institute of Medical Science, College of Medicine, The Catholic University of Korea, Seoul 06591, Republic of Korea; suky0215@catholic.ac.kr (Y.-S.S.); ektha40@catholic.ac.kr (D.-S.K.); elite@catholic.ac.kr (S.G.J.); 2 Department of Biomedicine & Health Sciences, College of Medicine, The Catholic University of Korea, Seoul 06591, Republic of Korea; 3 Division of Rheumatology, Department of Internal Medicine, Seoul St. Mary’s Hospital, College of Medicine, The Catholic University of Korea, Seoul 06591, Republic of Korea; elwin84@catholic.ac.kr

**Keywords:** systemic lupus erythematosus, rebamipide, podocyte, animal model

## Abstract

Systemic lupus erythematosus (SLE) is a prototypical autoimmune disease that affects various organs, including the kidneys. Despite recent advancements, effective treatment options for renal involvement in SLE remain limited. Rebamipide, originally developed as a gastroprotective agent, has been reported to exert immunomodulatory effects in rheumatic diseases. Here, we aimed to evaluate the therapeutic potential of rebamipide in SLE using an animal model and to elucidate its mechanisms of action. We administered rebamipide or vehicle control to lupus-prone MRL/*lpr* mice and evaluated its efficacy on lupus-like phenotypes, including renal manifestations and immune cell profiles. Additionally, we investigated potential therapeutic mechanisms through in vitro treatment of murine immune cells and podocytes with rebamipide. Oral administration of rebamipide in lupus-prone mice significantly reduced kidney size, weight, and histopathological inflammation. Among circulating immune cell subsets, only regulatory T cells were significantly increased by rebamipide. In vivo treatment with rebamipide enhanced the expression of podocyte structural proteins, such as Synaptopodin, in kidney tissues, accompanied by the recovery of antioxidative factors, including nuclear factor erythroid 2-related factor 2 (Nrf2). Similarly, in vitro treatment of murine immune cells and podocytes with rebamipide replicated its immunoregulatory and antioxidative effects. Rebamipide is proposed as a potential therapeutic candidate for managing renal involvement in SLE through its antioxidative effects on podocytes.

## 1. Introduction

Systemic lupus erythematosus (SLE) is a prototypical autoimmune disease characterized by the formation of autoantibodies, deposition of immune complexes, and subsequent inflammatory processes in target organs, leading to tissue damage [1]. During the pathophysiological progression of SLE, various immune cells, including dendritic cells, autoreactive T and B cells, and proinflammatory cytokines such as type I interferons (IFNs) and B cell-activating factor (BAFF), play key roles in disease development [2]. SLE primarily affects wome of reproductive age and can involve multiple organs, including the joints, skin, heart, and kidneys [1].

Among the various organ manifestations of SLE, renal involvement is one of the most clinically significant complications. Lupus nephritis, characterized by immune complex-mediated glomerulonephritis and subsequent podocyte injury, is the predominant form of renal involvement [3]. Severe cases may progress to end-stage renal disease, affecting approximately 10–30% of patients and significantly impacting survival [4]. Given these serious consequences, numerous therapeutic strategies have been explored, targeting disease-related cytokines such as IFNs and BAFF, in addition to general immunosuppressants [4]. However, neutralizing antibodies against IFNs and BAFF have demonstrated limited efficacy in treating lupus nephritis [5,6]. Meanwhile, the novel calcineurin inhibitor voclosporin has shown promising results, yet its availability remains limited in many countries [7]. Thus, there remains a critical need for effective therapeutic options for SLE.

Rebamipide is a mucosal protective agent primarily used for the treatment of gastric diseases, such as gastritis and gastric ulcers. Beyond its mucoprotective mechanisms, including the enhancement of prostaglandin and mucin production, rebamipide has also demonstrated anti-inflammatory properties through its antioxidative effects and modulation of T and B cell activity [8,9]. Recent preclinical studies using animal models have reported their therapeutic efficacy in several rheumatic diseases, including rheumatoid arthritis, osteoarthritis, and Sjögren’s syndrome [10,11,12]. Furthermore, some clinical studies have suggested its potential efficacy in autoinflammatory diseases, such as Behçet’s disease [13].

Given its anti-inflammatory properties, this study aimed to evaluate the therapeutic efficacy of rebamipide in SLE using lupus-prone mouse models. The results of the present study demonstrate that the oral administration of rebamipide reduces renal inflammation in lupus-prone mice. Specifically, rebamipide increased the population of circulating regulatory T (Treg) cells and restored the structural integrity of podocytes within glomeruli, accompanied by an upregulation of antioxidative factors. Similarly, in vitro treatment of murine podocytes and immune cells with rebamipide replicated these immunoregulatory and antioxidative effects.

## 2. Results

### 2.1. Effects of In Vivo Rebamipide Treatment on Lupus-like Phenotypes in a Mouse Model

**Figure 1:**
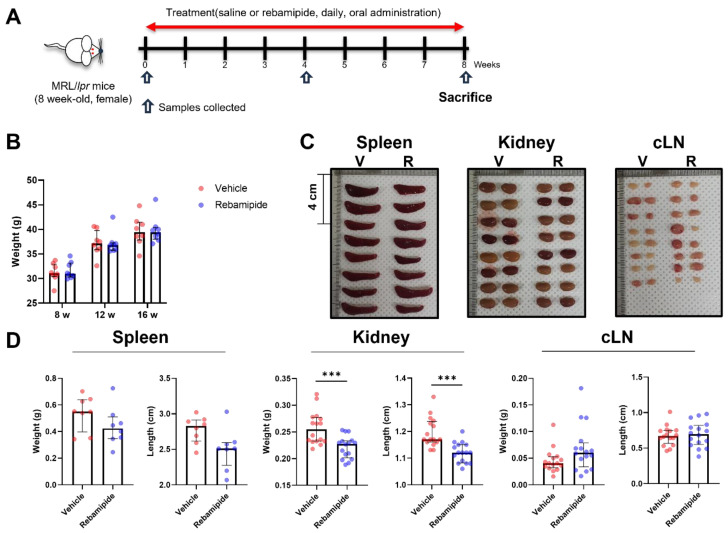
Effects of in vivo rebamipide treatment on lupus-like phenotypes in a mouse model. (**A**) Schematic illustration of the in vivo treatment of lupus-prone mice with rebamipide. Female MRL/*lpr* mice (8 weeks old, *n* = 8 per group) were administered rebamipide (5 mg/kg) or normal saline orally every day for 8 weeks. Samples were collected at 8, 12, and 16 weeks of age. All mice were euthanized at 16 weeks of age. (**B**) Body weight changes in each experimental group during in vivo treatment. (**C**) Representative gross images of spleens, kidneys, and cervical lymph nodes (cLNs) from 16-week-old mice treated with vehicle (V) or rebamipide (R). (**D**) Weights and lengths of spleens, kidneys, and cLNs from 16-week-old mice treated with vehicle or rebamipide. Data are presented as medians with interquartile ranges. Statistical analyses were performed using two-way ANOVA (**B**) and the Mann–Whitney test (**D**). *** *p* < 0.001.

To evaluate the therapeutic efficacy of rebamipide in lupus-like conditions, we utilized MRL/Mp-*Fas^lpr^* (MRL/*lpr*) mice as an animal model for SLE. MRL/*lpr* mice are widely recognized as lupus-prone due to the *Fas^lpr/lpr^* mutation [14]. These mice develop lupus-like phenotypes, including lymphadenopathy, splenomegaly, dermatitis, autoantibody production, immune complex deposition, and proteinuria caused by glomerulonephritis [15]. These manifestations peak at approximately 15–16 weeks of age. We administered rebamipide or vehicle to 8-week-old female MRL/*lpr* mice (*n* = 8 per group) for 8 weeks, after which blood and urine samples were collected, and the mice were euthanized for tissue pathology evaluation (Figure 1A). Body weight changes did not differ between the two groups (Figure 1B). However, the kidneys of the rebamipide-treated mice exhibited significantly reduced weight and length compared to those of vehicle-treated mice (Figure 1C,D). A similar trend was observed in spleens, whereas cervical lymph nodes showed no significant difference. These findings suggest that rebamipide treatment leads to clinical improvement in renal involvement in lupus-prone mice.

### 2.2. Effects of In Vivo Rebamipide Treatment on Immune Cell Subsets and Serologic Phenotypes

**Figure 2:**
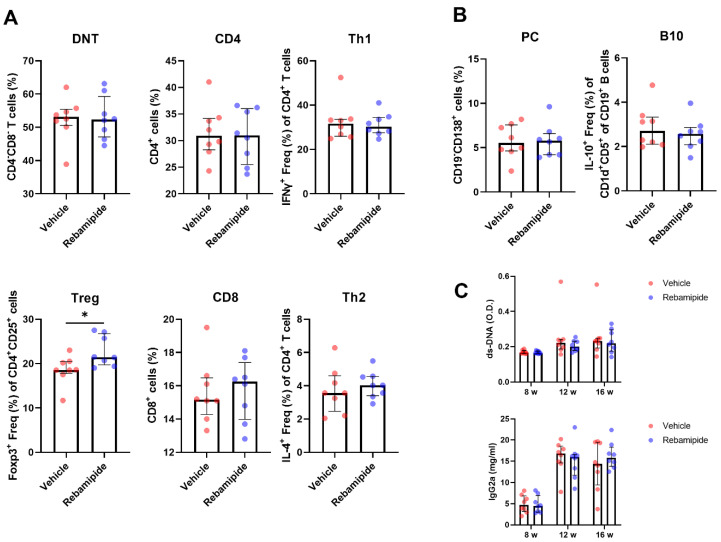
Effects of in vivo rebamipide treatment on immune cell subsets and serologic phenotypes. (**A**,**B**) Immune cell subsets in splenocytes from 16-week-old mice treated with vehicle or rebamipide, analyzed by flow cytometry. (**A**) Percentages of double negative T (DNT, CD4^−^CD8^−^) cells, CD4^+^ T cells, T helper 1 (Th1, CD4^+^IFNg^+^) cells, regulatory T (Treg, CD4^+^CD25^+^Foxp3^+^) cells, CD8^+^ T cells, and T helper 2 (Th2, CD4^+^IL-4^+^) cells. (**B**) Percentages of plasma B cells (PC, CD19^−^CD138^+^) and IL-10-producing B cells (B10, CD19^+^CD1d^+^CD5^+^IL-10^+^). (**C**) Serum levels of anti-dsDNA antibodies and immunoglobulin G2a (IgG2a) in the two groups of mice at 8, 12, and 16 weeks of age, determined by ELISA. Data are presented as medians with interquartile ranges. Statistical analyses were performed using the Mann–Whitney test (**A**,**B**) and two-way ANOVA (**C**). * *p* < 0.05.

Next, we evaluated the effects of rebamipide on various immune cell subsets using flow cytometric analysis of splenocytes isolated from mice. Double-negative T (DNT) cells represent a distinct T cell subset characterized by the absence of both CD4 and CD8 surface markers [16]. These cells are known to increase in MRL/*lpr* mice and correlate with the severity of lupus-like phenotypes [17]. In this study, rebamipide did not induce significant changes in the population of DNT cells, CD4⁺ T cells, CD8⁺ T cells, Th1 cells, or Th2 cells. However, Treg cells were significantly increased following rebamipide treatment (Figure 2A). Among B cell subsets, plasma B cells (PCs) and IL-10-producing B (B10) cells play key roles in autoantibody formation and immune regulation, respectively. In vivo rebamipide treatment did not significantly alter the population of these B cell subsets (Figure 2B). Consistent with these findings, serum levels of anti-dsDNA antibodies and IgG2a were not significantly different between the two groups (Figure 2C). Taken together, these results indicate that Treg cells are the only immune cell subset significantly affected by rebamipide treatment in lupus-prone mice.

### 2.3. Effects of In Vivo Rebamipide Treatment on Renal Manifestations

**Figure 3:**
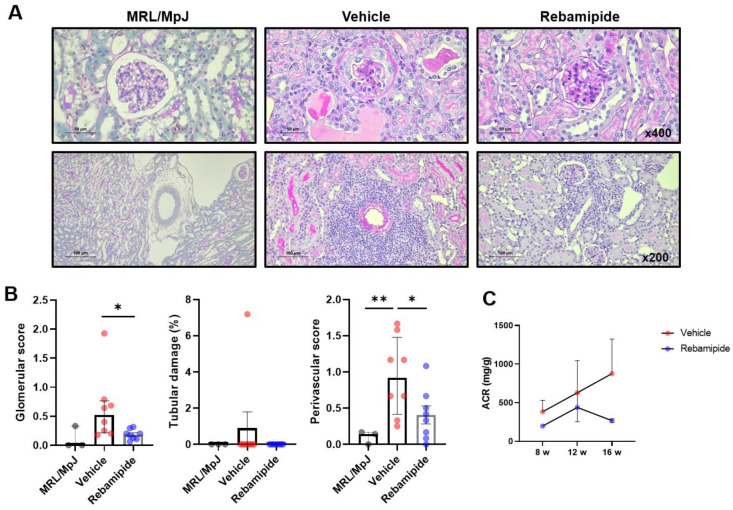
Effects of in vivo rebamipide treatment on renal manifestations. (**A**) Representative images of PAS-stained kidney tissues from mice treated with vehicle or rebamipide, showing glomerular (top) and perivascular (bottom) pathology. (**B**) Scores for glomerular pathology, tubular damage, and perivascular cell infiltration. (**C**) Urinary albumin/creatinine ratio (ACR) from each group of mice at 8, 12, and 16 weeks of age, measured by ELISA. Data are presented as medians with interquartile ranges. Statistical analyses were performed using the Mann–Whitney test (**B**) and two-way ANOVA (**C**). * *p* < 0.05, ** *p* < 0.01.

Subsequently, we assessed the therapeutic effects of rebamipide on renal phenotypes in lupus-prone mice. Histopathological examination of kidney tissues revealed that rebamipide-treated mice exhibited significantly reduced glomerular and perivascular inflammation compared to vehicle-treated mice (Figure 3A,B). Additionally, the urinary albumin-to-creatinine ratio, an indicator of proteinuria severity, showed a decreasing trend in rebamipide-treated mice compared to vehicle-treated mice (Figure 3C). Taken together with gross morphological findings (Figure 1C), these renal pathological findings suggest the therapeutic potential of rebamipide in lupus nephritis.

### 2.4. Expression Levels of Functional Proteins in Podocytes of Lupus-Prone Mice

**Figure 4:**
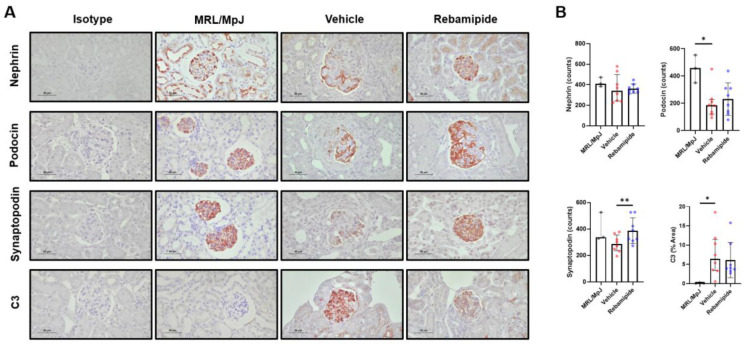
Expression levels of functional proteins in podocytes of lupus-prone mice. (**A**) Representative images of immunohistochemical staining of Nephrin, Podocin, Synaptopodin, and C3 in kidney tissues from 16-week-old MRL/*lpr* mice treated with vehicle or rebamipide (original magnification ×400). (**B**) Absolute counts of stained cells (Nephrin, Podocin, Synaptopodin) or percentages of stained area (C3). Data are presented as medians with interquartile ranges. Statistical analyses were performed using the Mann–Whitney test (**B**). * *p* < 0.05, ** *p* < 0.01.

Podocytes are specialized epithelial cells that constitute the renal glomerulus [18]. These cells play a critical role in the primary function of the glomerulus—selective filtration—by forming unique structural components such as foot processes and the slit diaphragm [18]. Podocin, Nephrin, and Synaptopodin are key functional proteins that maintain the structural integrity of podocytes [19]. In kidney tissues from patients with lupus nephritis, inflammation leads to podocyte injury by reducing the expression of these proteins and compromising the integrity of their functional structures, ultimately resulting in proteinuria [20]. Given this pathophysiological mechanism, we investigated whether rebamipide treatment could mitigate podocyte injury in lupus-prone mice. Histological analysis of murine kidney tissues using IHC staining for podocyte functional proteins and complement deposition revealed a significant restoration of Synaptopodin expression, along with a similar trend for Nephrin and Podocin (Figure 4A,B). Meanwhile, complement deposition, as indicated by IHC staining for C3, exhibited a decreasing trend (Figure 4A,B). These findings suggest that rebamipide may alleviate podocyte injury in the kidneys of lupus-prone mice.

### 2.5. Expression Levels of Oxidative Stress-Related Factors in Mouse Kidneys

**Figure 5:**
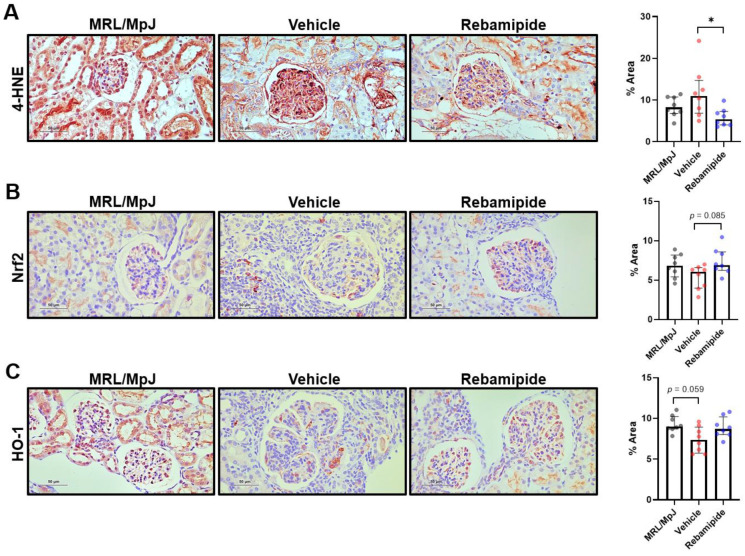
Expression levels of oxidative stress-related factors in mouse kidneys. Kidney tissues were collected from 16-week-old MRL/MpJ and MRL/*lpr* mice treated with vehicle or rebamipide (*n* = 7 per group). (**A**) Representative images (left panel, original magnification ×400) and percentages of stained area (right panel) for immunohistochemical staining of 4-hydroxy nonenal (4-HNE). (**B**) Representative images (left panel, original magnification ×400) and percentages of stained area (right panel) for nuclear factor erythroid 2-related factor 2 (Nrf2). (**C**) Representative images (left panel, original magnification ×400) and percentages of stained area (right panel) for heme oxygenase-1 (HO-1). Data are presented as medians with interquartile ranges. Statistical analyses were performed using one-way ANOVA. * *p* < 0.05.

Our previous findings suggest that rebamipide treatment in lupus-prone mice mitigated renal inflammation but exerted limited direct effects on immune cells, except for Treg cells. Given the known antioxidative properties of rebamipide, we investigated whether its therapeutic efficacy in renal phenotypes is mediated by antioxidative activity. Chronic inflammatory conditions in renal pathologies have been reported to induce podocyte injury through increased oxidative stress [21]. To assess oxidative stress levels in the kidneys of lupus-prone mice, we performed IHC staining for 4-hydroxynonenal (4-HNE), a widely used oxidative stress marker [22]. 4-HNE can activate antioxidative responses mediated by various transcription factors and enzymes, including Nrf2 and heme oxygenase-1 (HO-1) [22]. Kidney tissues from MRL/*lpr* mice exhibited an increasing trend in 4-HNE expression compared to those from MRL/MpJ mice, which served as healthy controls (Figure 5A). Additionally, in vivo rebamipide treatment reduced 4-HNE expression levels in the kidney tissues of lupus-prone mice (Figure 5A). Consistent with these findings, the expression levels of antioxidative factors, such as Nrf2 and HO-1, which were decreased in the kidneys of lupus-prone mice, were restored following rebamipide treatment (Figure 5B,C). Collectively, these findings suggest that the kidneys of lupus-prone mice experience oxidative stress-induced damage and that rebamipide treatment may enhance antioxidative responses.

### 2.6. Effects of In Vitro Rebamipide Treatment on Murine Podocytes and Immune Cells

**Figure 6:**
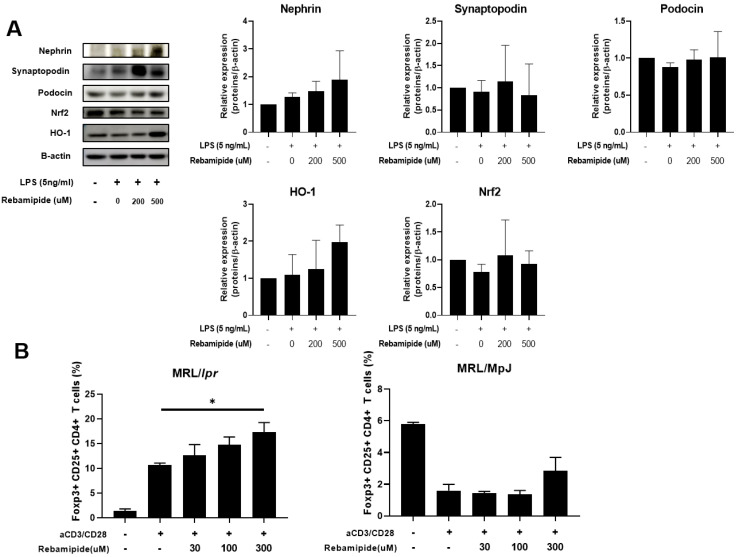
Effects of in vitro rebamipide treatment on murine podocytes and immune cells. (**A**) Primarily, cultured podocytes, isolated from mouse kidneys as described in the Methods, were co-treated with LPS (5 ng/mL) and varying concentrations of rebamipide (0 µM, 200 µM, 500 µM) for 24 h. The expression levels of Nephrin, Synaptopodin, Podocin, Nrf2, and HO-1 were analyzed by Western blot. (**B**) T cells were isolated using CD4 microbeads from the splenocytes of MRL/MpJ and MRL/*lpr* mice. T cells were stimulated with anti-CD3/CD28 for 2 h and then treated with graded doses of rebamipide for 5 days. The percentage of Treg cells among CD4⁺ T cells is shown. Data represent three independent experiments and are presented as mean ± SEM. Statistical analyses were performed using one-way ANOVA. * *p* < 0.05.

To investigate whether the antioxidative effects of rebamipide directly affect podocytes, the primary target cells in lupus nephritis, we isolated and cultured podocytes from mouse kidneys. These cells were exposed to increasing oxidative stress using lipopolysaccharide (LPS) and subsequently treated with graded doses of rebamipide. The expression levels of structural proteins and antioxidative factors were analyzed by Western blot. Primary mouse podocytes exhibited decreased expression of structural proteins, including Nephrin, Synaptopodin, and Podocin, following LPS-induced oxidative stress (Figure 6A). In vitro treatment with rebamipide showed a trend toward restoring the expression of these proteins, with the most prominent effect observed in Synaptopodin, consistent with the in vivo results from lupus-prone mice (Figure 6A). Similarly, the expression levels of antioxidative factors, such as Nrf2 and HO-1, were reduced following LPS-induced injury but were restored by rebamipide treatment (Figure 6A). In vitro treatment of human podocytes with rebamipide also produced similar results (Supplementary Figure S1). Finally, we investigated whether rebamipide promotes the differentiation of Treg cells from mouse CD4⁺ T cells. CD4⁺ T cells isolated from MRL/*lpr* mice were treated with graded doses of rebamipide, which resulted in a significant, dose-dependent increase in Treg cell differentiation (Figure 6B). However, this effect was not observed in CD4⁺ T cells isolated from MRL/MpJ mice (Figure 6B). Taken together, these in vitro findings suggest that rebamipide exerts direct antioxidative and immunomodulatory effects on murine podocytes and T cells, respectively.

## 3. Discussion

In the present study, oral administration of rebamipide ameliorated renal pathology in lupus-prone MRL/*lpr* mice. The renal manifestations improved by rebamipide treatment included proteinuria and inflammatory infiltration in the renal glomerulus. Histopathological analysis of kidney tissues revealed that rebamipide preserved podocyte integrity, reduced oxidative stress, and upregulated antioxidative factors such as Nrf2 and HO-1. The immunomodulatory effects of oral rebamipide administration on immune cells were limited in lupus-prone mice; however, increased differentiation of Treg cells was observed in both in vivo and in vitro experiments using MRL/*lpr* mice. Additionally, the results from in vitro experiments using both mouse and human cells suggest that podocytes are direct targets of the antioxidative effects of rebamipide.

Rebamipide has been reported to exert immunomodulatory effects in various animal models of autoimmune rheumatic diseases. A previous study using NFS/*sld* mice, an animal model of Sjögren’s syndrome, demonstrated that rebamipide significantly reduced autoreactive T cell proliferation and Th1 cytokine production [10]. Another study using NOD/ShiLtJ mice, another Sjögren’s syndrome model, showed that rebamipide ameliorated disease progression by decreasing T helper 17 (Th17) cells while increasing regulatory B cells, such as B10 cells [23]. Several studies on rheumatoid arthritis, using different animal models, including collagen-induced arthritis (CIA) in DBA/1J mice and zymosan A-induced arthritis in SKG mice, demonstrated that rebamipide could attenuate arthritis by suppressing Th1, Th2, and Th17 cells while increasing Treg cells [11,24]. Consistent with these findings, our results showed an increased population of Treg cells in lupus-prone mice following oral administration of rebamipide. The results from in vitro experiments using CD4⁺ T cells isolated from MRL/*lpr* mice further support these findings. However, in the present study, systemic administration of rebamipide did not affect other immune cell subsets, including Th1, Th2, DNT cells, plasma cells, and B10 cells. The discrepancy between our findings and those of studies on other autoimmune diseases may stem from differences in the characteristics and severity of the animal models used for each disease. Since SLE is one of the most severe autoimmune diseases in terms of disease burden and prognosis, MRL/*lpr* mice, one of the most widely used SLE models, also exhibit a relatively more severe phenotype compared to other autoimmune disease models. Given these considerations, rebamipide may exert modest immunomodulatory effects in lupus conditions, rather than broadly inhibiting pro-inflammatory immune responses. This suggests that rebamipide may be more suitable as an adjunctive treatment option for SLE rather than as a primary therapeutic modality.

Podocytopathies are kidney diseases caused by podocyte injury, leading to proteinuria and associated clinical symptoms. Hemodynamic and metabolic insults can induce podocytopathies in the form of hypertensive nephropathy and diabetic nephropathy, respectively [25]. Additionally, immune-mediated injury can also contribute to podocytopathies, such as membranous nephropathy and IgA nephropathy [25]. Lupus nephritis is another example of an immune-mediated podocytopathy [25]. A previous study reported a correlation between podocyte injury and oxidative stress biomarkers in kidney tissues of lupus nephritis patients [26]. Furthermore, in vivo experiments using MRL/*lpr* mice demonstrated that a deficiency of structural podocyte proteins, such as Nestin, exacerbated proteinuria by increasing mitochondrial dysfunction and oxidative stress [27]. In our study, we observed increased oxidative stress in the kidney tissues of lupus-prone MRL/*lpr* mice. Rebamipide treatment reduced oxidative stress and restored the expression of podocyte structural proteins while also upregulating antioxidative factors such as Nrf2 and HO-1. These findings are consistent with previous studies using animal models of rheumatoid arthritis, which demonstrated the induction of Nrf2 and HO-1 following rebamipide administration [11]. Among several structural podocyte proteins, Synaptopodin was the most affected by rebamipide treatment in both in vivo and in vitro experiments. Synaptopodin is a key structural protein that maintains the foot processes of podocytes, which are essential for the integrity of the glomerular filtration barrier in the kidneys [19]. The prominent effects of rebamipide on Synaptopodin expression further support its role in reducing proteinuria and improving renal phenotypes in lupus-prone mice.

Our findings suggest that rebamipide’s renoprotective effects in this lupus nephritis model primarily stem from direct cellular protection and the inhibition of oxidative stress, rather than a significant modulation of systemic immunological abnormalities. This mechanistic insight indicates a potential for rebamipide’s application beyond diseases predominantly driven by immune dysfunction. Supporting this broader applicability, previous studies have demonstrated rebamipide’s efficacy in various drug-induced nephrotoxicity models, such as those involving cisplatin, NSAIDs, methotrexate, and gentamicin [28,29,30,31]. In these contexts, rebamipide consistently improved kidney function by modulating key antioxidant parameters, including activating Nrf2 and HO-1 [30,31], reducing malondialdehyde levels [28,30,31], and increasing superoxide dismutase and glutathione activities [30,31]. These collective observations underscore rebamipide’s therapeutic promise in diverse forms of kidney damage where oxidative stress and cellular injury are central pathological features.

Another critical consideration for the clinical translation of rebamipide in renal disease concerns the long-term effects of its discontinuation. While long-term data specifically on renal disease outcomes following rebamipide cessation are currently limited, rebamipide has a well-established safety profile from its continuous use as a gastrointestinal protective agent [32]. Nevertheless, future comprehensive studies are essential to determine whether renal disease might worsen upon discontinuation of rebamipide treatment, providing crucial information for clinical practice guidelines.

Rebamipide has been approved and is already prescribed as a gastroprotective agent. However, the clinical dose of rebamipide in human patients is relatively lower than the dose administered to mice in this study. Therefore, the appropriate dosage of rebamipide for reducing proteinuria in lupus nephritis should be carefully evaluated. A previous study using CIA mouse models of rheumatoid arthritis demonstrated that high doses of rebamipide (30 mg/kg) exacerbated arthritis severity rather than alleviating it [33]. Consistent with this, in our study, higher doses of rebamipide (10 mg/kg) resulted in reduced or diminished efficacy on lupus-like phenotypes in animal models. Metabolomic analyses of rebamipide treatment using mass spectrometry suggested that the dose-dependent variation in efficacy may be attributed to the differential expression of metabolites, such as corticosterone [33]. Based on these findings, disease-specific dosing strategies for rebamipide should be established before considering its use in human patients.

There are several limitations to this study that should be discussed. First, although this study demonstrated the efficacy of rebamipide in mitigating podocyte injury and oxidative stress, it does not establish a direct causal relationship between these factors. The results suggest only an association, and further research is needed to elucidate the precise mechanisms underlying the therapeutic effects of rebamipide in lupus nephritis. Second, this study did not investigate the specific receptors and intracellular signaling pathways in podocytes and immune cells that may be affected by rebamipide treatment. Previous studies have suggested that rebamipide can regulate the expression of key inflammatory factors such as the nuclear factor kappa-light-chain-enhancer of activated B cells and the signal transducer and activator of transcription 3 in proinflammatory T cell subsets [10,11]. However, in this study, these immune cell subsets did not show significant changes following rebamipide treatment. We proposed Nrf2 and HO-1 as potential antioxidative pathways through which rebamipide exerts its effects on podocytes, but the exact mechanisms remain to be fully elucidated. Third, this study utilized only a single SLE model—spontaneous lupus-prone MRL/*lpr* mice. Although this is one of the most widely used animal models of SLE, it does not fully represent the diverse phenotypic spectrum of the disease. Furthermore, the aggressive disease phenotype in this model may have contributed to discrepancies in the effects of rebamipide on immune cell subsets compared to previous reports. Therefore, future studies using different SLE models are warranted to further validate the effects of rebamipide in lupus conditions.

## 4. Materials and Methods

### 4.1. Animals

MRL/*lpr* mice (female, 8 weeks old) and MRL/MpJ mice (female, 8 weeks old) were purchased from SLC Inc. (Hamamatsu, Shizuoka, Japan). Mice were randomly divided into two groups, vehicle— or rebamipide (Ace Biopharma Inc., Seoul, Republic of Korea) —treated. Rebamipide was dissolved in 10% DMSO and 90% saline. MRL/*lpr* mice were orally treated with 5 mg/kg of rebamipide every day for 8 weeks. The vehicle control group received the same volume of 10% DMSO. After 8 weeks of administration, the mice were sacrificed. Serum was collected from peripheral blood and the kidneys and the spleens were collected. All animal experiments were performed in accordance with the Laboratory Animals and the Guidelines and Policies for Rodent experiments provided by the Institutional Animal Care and Use Committee in College of Medicine, The Catholic University of Korea (approval number: 2021-0257-02).

### 4.2. Primary Mouse Podocyte Isolation

After transcardiac perfusion with Dynabeads M-450 Epoxy (Thermo Fisher, Waltham, MA, USA) suspended in PBS, the kidneys were isolated from 8–12 week-old C57BL/6 mice and minced. The kidneys were digested with collagenase A (Sigma-Aldrich, Burlington, MA, USA) and DNase I (Merck, Burlington, MA, USA) in HBSS at 37 °C for 30 min and subsequently gently pressed through a 100 µm cell strainer. Glomeruli containing Dynabeads were gathered by DynaMagTM-2 (Thermo Fisher), washed three times with HBSS (Thermo Fisher), and finally cultured the isolated glomeruli on Type-1 collagen-coated dishes in DMEM/F12 (Gibco, Waltham, MA, USA) containing 5% fetal bovine serum (FBS, Gibco) with 0.5% insulin-transferin-selenium, liquid media supplement (Gibco), and 1% penicillin-streptomycin (Gibco). The cells were incubated at 37 °C with 5% CO_2_ for 4–8 days and subcultured by removing the glomeruli using a magnet. After subculture, the cells were co-treated with LPS (5 ng/mL, Sigma-Aldrich) and rebamipide (0 µM, 200 µM, 500 µM) for 24 h.

### 4.3. Human Podocyte Cell Line Culture and Treatments

The human podocyte cell line (LY cell line) was purchased from the University of Bristol, UK. The cells were cultured at 33 °C in Roswell Park Memorial Institute (RPMI) 1640 (Gibco), containing 10% FBS and 1% penicillin-streptomycin. After cultivation to an 80–90% confluence, the podocytes were subcultured for 10–14 days under 5% CO_2_ at 37 °C to induce cell differentiation. The cells were co-treated with LPS (5 ng/mL) and rebamipide (0 µM, 100 µM, 200 µM, 500 µM) for 24 h.

### 4.4. Histologic Assessment of Kidneys

The kidneys, isolated from sacrificed mice and washed with phosphate-buffered saline, were fixed in 4% paraformaldehyde at 4 °C overnight, embedded in paraffin. Then, 4 µm kidney sections were stained with hematoxylin (Vector Labs, Newark, CA, USA) and eosin (Muto Pure Chemicals, Tokyo, Japan) (H&E) and periodic acid-schiff (PAS; Abcam, Cambridge, UK) reagents according to the manufacturer’s protocols. Kidney pathology was evaluated using the lupus nephritis classification system, as described elsewhere [34].

### 4.5. Immunohistochemistry (IHC)

Tissue sections of mouse kidneys were immersed in antigen retrieval solution (Dako, Nowy Sacz, Poland), then heated to 115 °C for 15 min in a pressure cooker (Bio SB, Santa Barbara, CA, USA). Endogenous peroxidase activity was quenched with 3% H_2_O_2_ (Sigma-Aldrich). IHC was performed using the VECTASTAIN ABC-HRP kit (Vector Labs). Tissues were first incubated with anti-guinea pig Nephrin (1:100) (Progen, Heidelberg, Germany), anti-rabbit Podocin (1:100) (Novusbio, Centennial, CO, USA), anti-rabbit Synaptopodin (1:1000) (Abcam), anti-rabbit-C3 (1:2000) (Abcam), anti-rabbit Nrf2 (1:100) (ABclonal, Woburn, MA, USA), anti-rabbit HO-1 (1:100) (Abcam), and anti-mouse 4-HNE (1:100) (R&D systems, Minneapolis, MN, USA) or isotype control antibodies (Santa Cruz, Dallas, TX, USA) overnight at 4 °C; they were then stained with biotinylated secondary antibodies and a streptavidin-peroxidase complex for 30 min. Staining was developed using DAB chromogen (Dako). Tissue sections were counterstained with hematoxylin QS (Vector Labs).

### 4.6. Flow Cytometry

Mouse spleens were minced in RPMI 1640 (Gibco) medium and filtered through a 40 µm cell strainer to prepare single-cell suspensions. For double negative T (DNT) cell surface staining, cells were stained with eFluor780-fixable viability dye (FVD) (eBioscience, San Diego, SA, USA), Pacific blue-conjugated anti-CD90.2 (BioLegend, San Diego, CA, USA), PerCP-Cy5.5-conjugated anti-CD4 (eBioscience), and PE-conjugated anti-CD8 (Biolegend). For the assessment of T helper 1 (Th1) and T helper 2 (Th2) cells, cells were stimulated with phorbol 12-myristate 13-acetate (PMA) (25 ng/mL, Sigma-Aldrich) and ionomycin (250 ng/mL, Sigma-Aldrich) with monensin-containing GolgiStop (BD, Franklin Lakes, NJ, USA) for 5 h. Cells were then stained with PerCP-Cy5.5 conjugated anti-CD4 (Biolegend). Subsequently, the cells were fixed and permeabilized using Cytofix/Cytoperm (BD). The fixed cells were stained with APC-conjugated IFNγ (Biolegend) and PE-conjugated IL-4 (BD). Similarly, for the assessment of Treg cells, cells were stained with PerCP-Cy5.5-conjugated anti-CD4 (Biolegend) and APC-conjugated anti-CD25 (Biolegend). Following fixing and permeabilization, cells were stained with PE-conjugated anti-FoxP3 (eBioscience) intracellularly. For plasma B cell (PC) surface staining, cells were stained with PerCP-Cy5.5-conjugated anti-CD19 (eBioscience) and PE-conjugated anti-CD138 (BD). For the assessment of B10 cells, cells were stimulated with PMA (Sigma-Aldrich) and ionomycin (Sigma-Aldrich) with monensin-containing GolgiStop (BD) for 5 h. Cells were harvested and stained with PerCP-Cy5.5-conjugated anti-CD19 (eBioscience), PE-conjugated anti-CD1d (eBioscience), and FITC-conjugated anti-CD5 (eBioscience). The cells were then fixed, permeabilized, and stained with APC-conjugated anti-IL-10 (eBioscience).

To identify the effects of rebamipide treatment on murine immune cells, T cells were isolated using CD4 microbeads (Miltenyi Biotec, NRW, Germany) from the splenocytes of MRL/MpJ and MRL/*lpr* mice. T cells were stimulated with anti-CD3/CD28 antibodies for 2 h and then treated with graded doses of rebamipide for 5 days. For intracellular staining, cells were stimulated with PMA (Sigma-Aldrich) and ionomycin (Sigma-Aldrich) with GolgiStop (BD) for 5 h. Then, cells were incubated with surface FVD (eBioscience), PerCP-Cy5.5-conjugated anti-CD4 (eBioscience), and APC-conjugated anti-CD25 (Biolegend). The cells were then fixed, permeabilized, and stained with PE-conjugated FoxP3 antibodies (eBioscience). Cells were analyzed using a BD LSRII Fortessa flow cytometer (BD), and the data were analyzed using FlowJo software (FlowJo LLC Inc., Ashland, OR, USA).

### 4.7. Enzyme-Linked Immunosorbent Assay (ELISA)

Collected sera from mice were assayed using the mouse anti-dsDNA IgG-specific ELISA kit (R&D systems) and anti-mouse immunoglobulin G2a (IgG2a) ELISA kit (Bethyl Laboratories, Montgomery, Tx, USA) according to the manufacturer’s instructions.

### 4.8. Western Blot

Cells were collected and lysed in RIPA buffer containing a Halt protease/phosphatase inhibitor cocktail (Thermo Fisher). The Bradford assay (Bio-Rad, Hercules, CA, USA) was used to measure the protein concentrations following the manufacturer’s instructions. Samples were boiled in 4x sample buffer for 5 min at 95 °C. The same amounts of protein (30 µg per sample) were separated by Bis-Tris gel (4–12%, Thermo Fisher) and subsequently transferred onto PVDF membranes and probed with the following antibodies: anti-Nephrin antibody (Progen), anti-Synaptopodin antibody (Abcam), anti-Podocin antibody (Novusbio), anti-Nrf2 antibody (Cell Signaling Technology, Danvers, MA, USA), anti-HO-1 antibody (Abcam), anti-glyceraldehyde-3-phosphate dehydrogenase (GAPDH, Abcam), and anti-beta actin antibody (β-actin, Santa Cruz). Subsequently, the membranes were incubated with horseradish peroxidase-conjugated goat anti-rabbit IgG or goat anti-mouse IgG (Thermo Fisher). Reactive proteins on the membrane were visualized by SuperSignal West Pico Chemiluminescent substrate (Thermo Fisher), and the membranes were analyzed using an Amersham Imager 600 (GE Healthcare, Chicago, IL, USA).

### 4.9. Statistics

Statistical analyses were performed using GraphPad Prism (version 8 for Windows; GraphPad Software, San Diego, CA, USA). Statistical significance was determined by the Mann–Whitney test, by one-way ANOVA with Tukey multiple comparison test, or by two-way ANOVA with Bonferroni post-test. *p* values of <0.05 were considered to be significant.

## 5. Conclusions

Effective therapeutic options for SLE remain an unmet clinical need. Although various biologics and small-molecule inhibitors targeting specific pathophysiological pathways of SLE are under development, their clinical application remains uncertain. The present study suggests that rebamipide, an agent already in clinical use, may serve as a novel therapeutic approach for renal involvement in SLE, based on experimental findings from animal models. Future studies are needed to further substantiate the potential of this agent as a treatment option for SLE.

## Data Availability

Data are contained within the article.

## Supplementary Materials

The following supporting information can be downloaded at https://www.mdpi.com/article/10.3390/ijms26125809/s1.
