# Supplementary material for: Rebamipide Attenuates Lupus Nephritis by Enhancing Antioxidative Defense in Podocytes: Evidence from a Lupus-Prone Mouse Model
Source: Int J Mol Sci. 2025 Jun 17;26(12):5809. doi: 10.3390/ijms26125809 (PMC12192773; doi:10.3390/ijms26125809)
Supplement: Supplementary file 1 [file ijms-26-05809-s001.zip › ijms-3574945-supplementary.pdf]

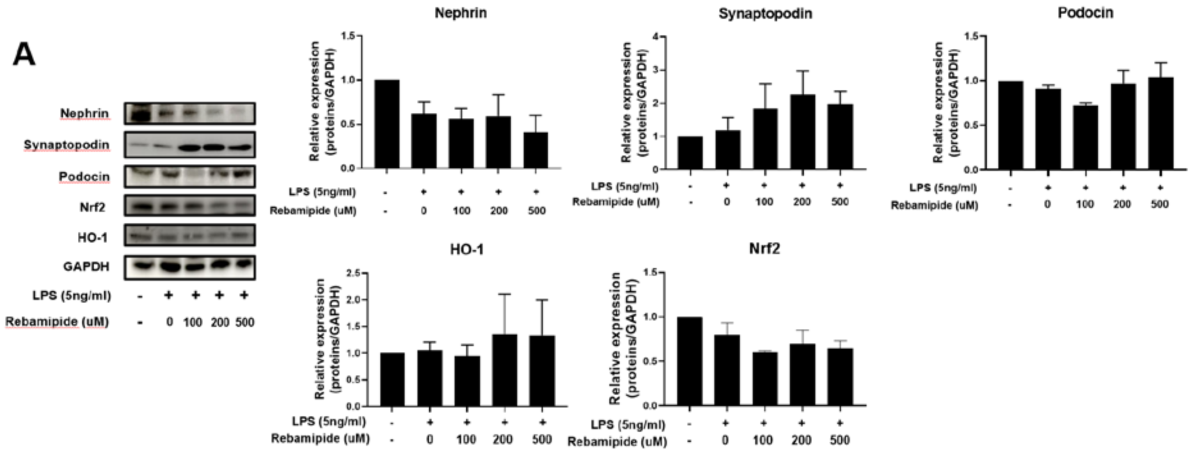

Supplementary Figure S1. Effects of *in vitro* rebamipide treatment on human podocytes. (**A**) Human podocytes (LY cell line) were cultured for 10–14 days under 5% CO_2 at 37°C to induce differentiation. The cells were co-treated with LPS (5 ng/ml) and graded doses of rebamipide for 24 hours. The expression levels of Nephrin, Synaptopodin, Podocin, Nrf2, and HO-1 were analyzed by western blot. Data represent three independent experiments and are presented as mean ± SEM. Statistical analyses were performed using one-way ANOVA.
